# Enhanced Capacitance of Hybrid Layered Graphene/Nickel Nanocomposite for Supercapacitors

**DOI:** 10.1038/srep32082

**Published:** 2016-08-24

**Authors:** Norsaadatul Akmal Mohd Zaid, Nurul Hayati Idris

**Affiliations:** 1 School of Ocean Engineering, Universiti Malaysia Terengganu 21030 Kuala Terengganu, Malaysia

## Abstract

In this work, Ni nanoparticles were directly decorated on graphene (G) nanosheets via mechanical ball milling. Based on transmission electron microscopy observations, the Ni nanoparticles were well dispersed and attached to the G nanosheet without any agglomerations. Electrochemical results showed that the capacitance of a G/Ni nanocomposite was 275 F g^−1^ at a current density of 2 A g^−1^, which is higher than the capacitance of bare G (145 F g^−1^) and bare Ni (3 F g^−1^). The G/Ni electrode also showed superior performance at a high current density, exhibiting a capacitance of 190 F g^−1^ at a current density of 5 A g^−1^ and a capacitance of 144 F g^−1^ at a current density of 10 A g^−1^. The equivalent series resistance for G/Ni nanocomposites also decreased. The enhanced performance of this hybrid supercapacitor is best described by the synergistic effect, i.e. dual charge-storage mechanism, which is demonstrated by electrical double layer and pseudocapacitance materials. Moreover, a high specific surface area and electrical conductivity of the materials enhanced the capacitance. These results indicate that the G/Ni nanocomposite is a potential supercapacitor.

The term “supercapacitor” is synonymous with “electrochemical capacitor” or “ultracapacitor”, has very attractive criteria including large power density, excellent cyclability and fast charging time^1^. Furthermore, a supercapacitor fulfil the inconsistency and bridging function between the common battery and a traditional dielectric capacitor in terms of specific energy and specific power^1^,^2^. Supercapacitors are broadly classified into two types that differ in their charge-storage mechanisms: the electrical double-layer capacitance (EDLC) and the pseudocapacitor^3^,^4^.

EDLC is based on carbonaceous materials that have high power density and low capacitance; its corresponding capacitance has a common value of less than 40 μF cm^−2^ of the real surface. This is an immense weakness for EDLC that needs improvement^5^. A pseudocapacitor is based on metals, metal oxides or conducting polymers; it can impart high specific capacitance but suffers from poor cyclability because of its low conductivity. Therefore, tremendous efforts have been made to enhance the energy and power densities of supercapacitors; one such effort hybridized EDLC and a pseudocapacitor to increase the electrochemical performance^6^,^7^,^8^.

Currently, graphene nanosheet known as new discovery; these nanosheets have a remarkable structure comprising a two-dimensional layered hexagonal lattice of carbon atoms and a large surface area of 2630 m^2^ g^−1^. G nanosheets are potential candidates for electrodes due to their high conductivity and good mechanical properties; these properties are comparable to or better than carbon nanotubes^9^,^10^,^11^. The specific capacitance of G as an electrode can achieve 135, 99, and 75 F g^−1^ in aqueous, organic electrolyte and ionic liquid electrolyte solutions, respectively^12^,^13^.

Alternatively, G-based nanocomposites have been combined with pseudocapacitive materials to enhance capacitance; research has been reported on G-based nanocomposites including G/ZnO^14^,^15^, G/NiO^16^,^17^,^18^ and G/RuO_2_^19^,^20^. Pseudocapacitive materials possess low conductivity and are characterized by slow electron transport; hybrid materials made from pseudocapacitive materials and G speed up electron transport through the supercapacitor electrode^15^,^21^,^22^. For example, Zhang *et al*.^11^ synthesized G and polyaniline nanofibre nanocomposites that exhibited a high capacitance of 480 F g^−1^ and good cyclability compared to bare G and bare polyaniline. Zhang *et al*.^15^ prepared G/ZnO using an ultrasonic spray pyrolysis technique and its capacitive behavior was improved. This hybrid supercapacitor had a synergistic effect resulting from the dual charge-storage mechanism comprising an electrical double layer and pseudocapacitance materials. Moreover, the high specific surface area and electrical conductivity of the materials enhance the capacitance. Here, we demonstrate a simple method that uses ball-milling to prepare G/Ni nanocomposite. This method could be extended to synthesize other G/metal nanocomposites in developing new materials for supercapacitors. Besides, the addition of Ni does not only contribute to the additional pseudocapacitance and to increase the EDLC of G, but also intensify the interaction between Ni and G to improve the electrochemical stability of the composites. This found to be an efficient way to improve the performance of the hybrid nanocomposites for supercapacitor^23^. Nevertheless, there is still much left to explore in the area of G-metal nanocomposites. With a simple and effective synthesis, a G/Ni nanocomposite with a high capacitance was obtained.

## Experimental section

### Synthesis of graphite oxide (GO) and G

GO was prepared using Hummer’s method according to previously reported procedures^24^. Specifically, 1 g of natural graphite (Sigma-Aldrich) was added to 50 mL of concentrated H_2_SO_4_ (Sigma-Aldrich) and stirred in an ice-water bath. 6 g of KMnO_4_ (Sigma-Aldrich) was added gradually, and the resultant mixture was stirred at room temperature for 1 h. Next, 80 mL of deionized (DI) water was slowly added while increasing the temperature to 90 °C. Then, 200 mL of DI water and 6 mL of 30% H_2_O_2_ (Merck) were added dropwise; the colour of the solution turned from dark brown to yellow. The precipitated of GO was segregated by centrifugation, washed several times with 5% HCl (Merck) and acetone (Emsure) and dried at 65 °C for 12 h under vacuum. Subsequently, 0.6 g of GO was dispersed into 200 mL of ethanol and then sonicated for 1 h. Under effective stirring at 60 °C, 10 mL of N_2_H_4_ (Sigma-Aldrich) was added dropwise to the mixture. The generated solid was separated by centrifugation and washed several times with DI water and ethanol. Finally, the as-prepared G was dried at 65 °C for 12 h under vacuum.

### Synthesis of graphene/nickel nanocomposite

A G/Ni nanocomposite was prepared by ball-milling G and Ni nanoparticle (Sigma-Aldrich) at a rotatory speed of 400 rpm for 20 h. The handlings of the samples were conducted in an MBraun Unilab glove box filled with high purity Ar atmosphere. The samples were put into a sealed stainless steel vial, together with hardened stainless steel balls. To avoid an increase in temperature during the process, the milling time was interrupted every 1 h for 6 min.

### Preparation of electrode and electrochemical measurements

The working electrode was prepared by dissolving 75 wt.% of G/Ni nanocomposite, 20 wt.% of carbon black (Aldrich) and 5 wt.% of polyvinylidene fluoride (Aldrich) in *N*-methyl-2-pyrrolidone (Sigma-Aldrich). A homogenous slurry was pasted onto titanium foil (1 × 1 cm^2^) and allowed to dry at 100 °C under vacuum.

Cyclic voltammetry (CV) analysis was performed using Autolab PGSTAT302 (Eco-chemie) in the potential range of −1.0 to 0.3 V. The capacitive behaviour of the G/Ni electrode was examined using a three-electrode system in 1 M potassium hydroxide (KOH) (Sigma-Aldrich) aqueous electrolyte. Rod platinum, a saturated calomel electrode and a G/Ni electrode were used as the counter electrode, reference electrode and working electrode, respectively.

The galvanostatic charge-discharge was conducted using a CHI 700E electrochemical workstation instrument. The specific capacitance (*C*_*sp*_) derived from galvanostatic curve, which can be calculated from equation (1) as follows:


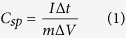


where *I* is the discharge current (A), *∆t* is the discharge time (s), *m* is the weight of the active material (g) and *∆V* is the potential range (V). Electrochemical impedance spectroscopy (EIS) was investigated in the frequency range between 0.01 Hz and 50 kHz at 10 mV.

### Materials characterization

The phase of the G/Ni nanocomposite was characterized by X-ray diffraction (XRD) using the Rigaku Miniflex II with monochromatic Cu Kα radiation (λ = 1.5406 Å). The composition of the G/Ni nanocomposite was estimated using a Mettler-Toledo TGA/DSC 1 Star^e^ System at a heating rate of 10 °C min^−1^ from room temperature to 600 °C in air. The morphology of the sample was examined using scanning electron microscopy (SEM; JEOL JSM-6360LA) and transmission electron microscopy (TEM; JEM 2100-F) with an accelerating voltage of 200 kV.

## Results and Discussion

### Structure and morphology analysis

**Figure 1:**
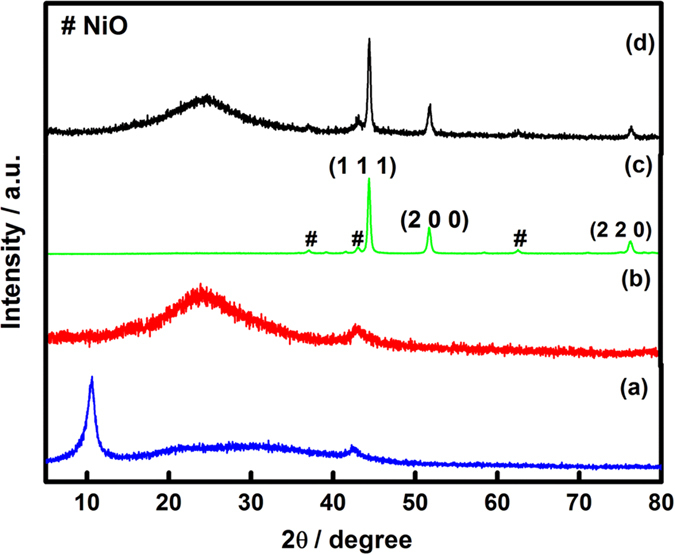
XRD spectra of (**a**) graphite oxide, (**b**) graphene, (**c**) nickel and (**d**) G/Ni nanocomposite.

Figure 1 shows the XRD patterns of GO, G, Ni and G/Ni nanocomposite. There are two sharp diffraction peaks at approximately 10.05° and 42.3° that correspond to GO. Diffraction peaks at approximately 26° and 43.5° were observed for G, indicating that GO was fully converted to G. The nickel nanoparticles received from Sigma-Aldrich contained NiO (JCPDS card number 87–712) as impurity. A G/Ni nanocomposite was successfully synthesized without any impurities; no chemical reactions took place during the ball-milling process. Furthermore, the broad character of the peak at 26° in the nanocomposite implies that G was distributed homogeneously without significant agglomeration or stacking^25^; this agreed with the TEM observation.

**Figure 2:**
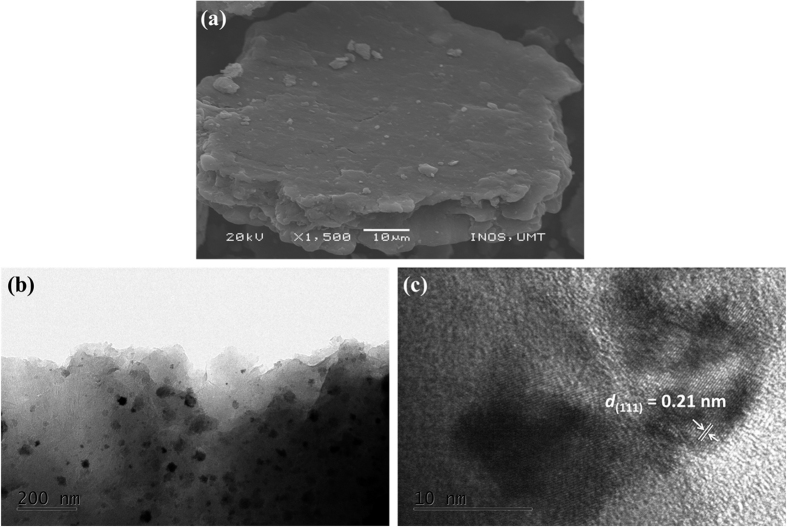
(**a**) SEM image, HRTEM images at (**b**) low magnification and (**c**) high magnification of G/Ni nanocomposite.

The morphology of G/Ni nanocomposites was observed with SEM and TEM, as shown in Fig. 2. Visualisation of G/Ni by SEM showed layers of a G nanosheet. Because the synthetic method was a simple ball-milling process between G and Ni nanoparticle, there was no chemical reaction between them. The TEM image showed that the Ni nanoparticles were distributed in the form of individual particles, approximately 26 nm in diameter. Figure 2(b) shows clusters of Ni nanoparticles at the surface of the G nanosheet. A transparent G nanosheet exhibited a 2D sheet structure comprising only a few layers—in some examples, the nanosheet only comprised one layer. Figure 2(c) shows a TEM image of a Ni nanoparticle with certain lattice planes (111) in focus and a corresponding *d*-spacing of 0.21 nm. The Ni nanoparticle was well dispersed and attached to the G nanosheet, indicating that ball milling could prevent Ni nanoparticles from agglomerating and could facilitate firm attachment to the G nanosheet.

**Figure 3:**
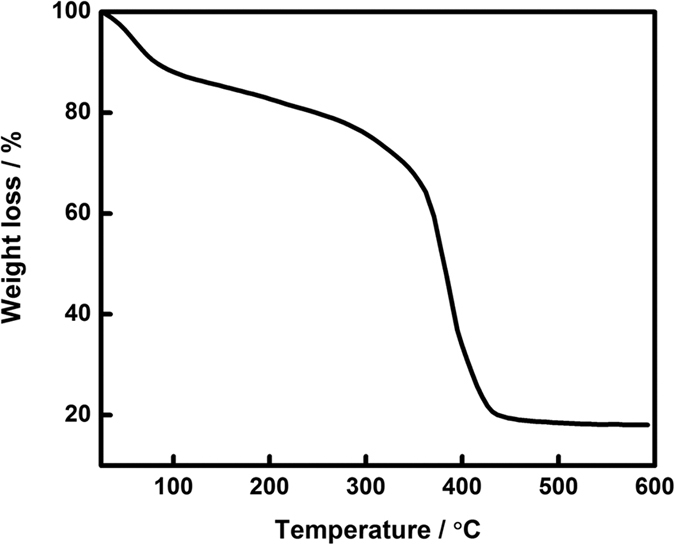
Thermogravimetric analysis (TGA) curve of G/Ni nanocomposite.

To estimate the amount of Ni in the G/Ni nanocomposite, TGA measurements were conducted at a heating rate of 10 °C min^−1^ from 25 °C to 600 °C in air. Figure 3 shows two steps of weight loss that occur between approximately 100 °C and 420 °C. After 420 °C, the TGA curve remains stable with no further weight loss. Based on this result, the amount of Ni in the G/Ni nanocomposite was estimated to be approximately 34 wt%.

### Electrochemical studies

**Figure 4:**
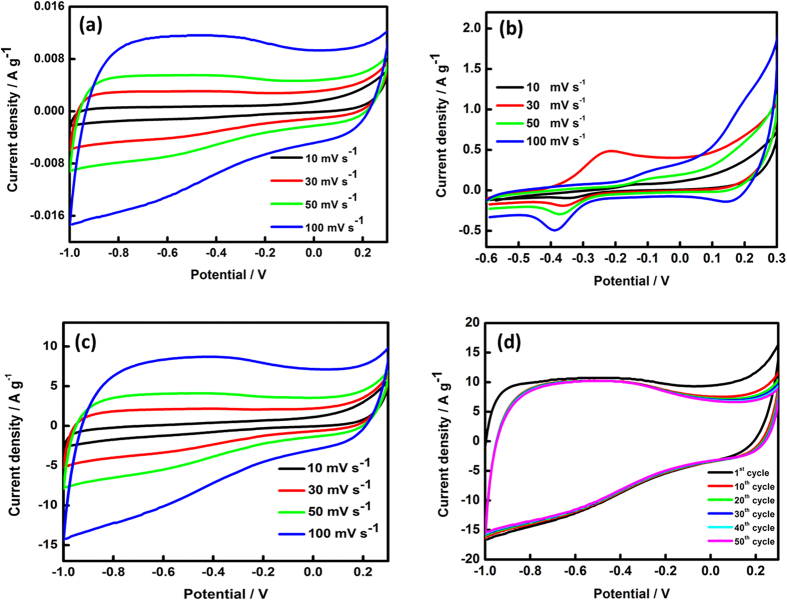
Cyclic voltammetry curves of (**a**) graphene, (**b**) nickel, (**c**) G/Ni nanocomposite at different scan rates and (**d**) G/Ni nanocomposite at selected cycles.

The performances of supercapacitor devices using G/Ni nanocomposites were analysed using CV, galvanostatic charge–discharge and EIS. CV can be used to examine the potential of a supercapacitor application by analysing two factors—different scan rates and cycle numbers. CV is also used to identify the oxidation and reduction peaks resulting from pseudocapacitance. Figure 4 shows comparative CVs of the G, Ni and G/Ni nanocomposites at different scan rates. Theoretically, the shape of the CV curve should be rectangular with an oblique angle and a narrow loop; this suggests that there is a large resistance and that low contact resistance affects the loop^1^. CVs of the G exhibited a rectangular shape; which is the typical curve for EDLCs and indicates that G exhibited good double-layer capacitive behaviour^26^. For a bare Ni nanoparticle, the CV shape was notably dissimilar from G. There were reduction and oxidation peaks during the anodic and cathodic sweeps; these peaks are related to Faradaic reactions, as shown in Fig. 4(b). At 10 mV s^−1^, a Ni nanoparticle showed a small redox peak at −0.12 and −0.32 V, whereas at 30 mV s^−1^ the redox peaks appeared at −0.25 and −0.35 V. At a scan rate of 50 mV s^−1^, the redox peak appeared at −0.37 and −0.17 V, whereas peaks at −0.39 and −0.18 V appeared at a scan rate of 100 mV s^−1^. The redox peaks were better defined the CV curve exhibited a larger area, suggesting a greater contribution of pseudocapacitance to the overall capacitance. The configuration of the curves indicated low capacitance behaviour and high contact capacitance. Moreover, for G/Ni nanocomposites, the curves (Fig. 4(c)) exhibited a so-called rectangular shape along the axis without obvious redox peaks even with the introduction of Ni nanoparticles^27^. This indicates that the G/Ni electrode had a high capability for supercapacitor application and a low equivalent series resistance (ESR)^28^. The shape of the CV curve was still markedly rectangular in shape, even at a high scan rate. The cycle number increased for electrodes, indicating an excellent high rate performance as shown in Fig. 4(d). The interaction between G and Ni nanoparticles produced a better electrochemical performance because of the pseudocapacitance arising from Ni sites and the EDLC contribution by the G.

**Figure 5:**
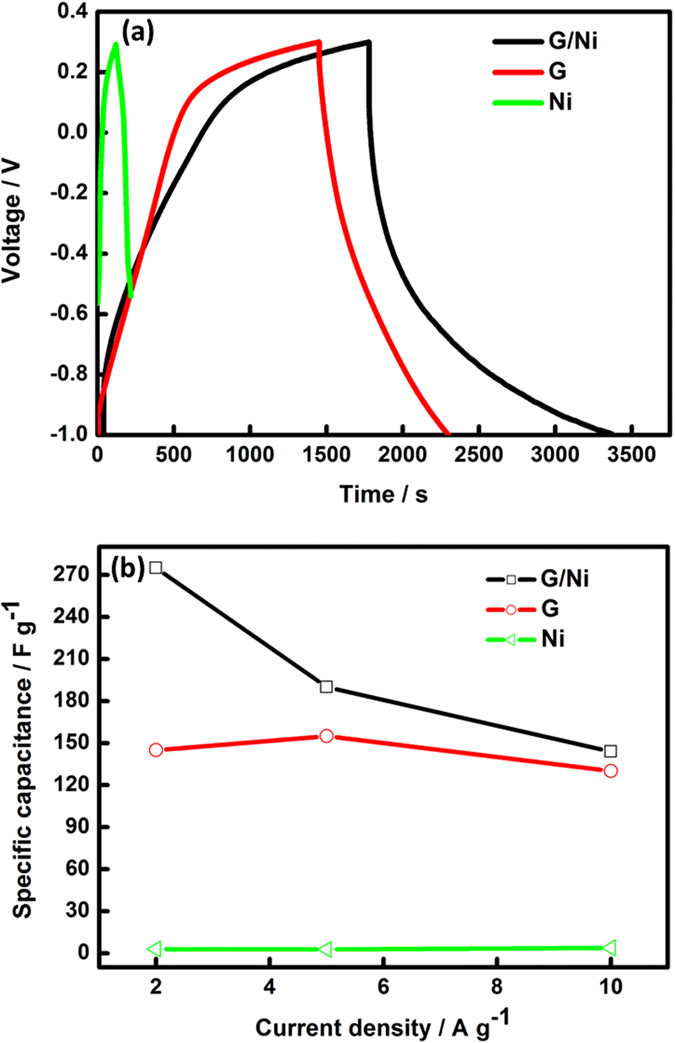
(**a**) Galvanostatic charge-discharge curves at current density of 2 A g^−1^ and (**b**) specific capacitance as a function of current density for graphene, nickel nanoparticle and G/Ni nanocomposite.

The specific capacitances of the G, Ni and G/Ni nanocomposites were calculated from the discharge slopes of the galvanostatic charge–discharge curves. Maximum capacitances of 275, 145 and 3 F g^−1^ at a current density of 2 A g^−1^ were obtained for the G/Ni nanocomposite, bare G and bare Ni, respectively. Moreover, the specific capacitance values for a G/Ni nanocomposite at higher current densities of 5 and 10 A g^−1^ were 190 and 144 F g^−1^, respectively. For G, the capacitance values were 155 and 130 F g^−1^, whereas for Ni nanoparticles, the capacitance values were 2.7 and 3.9 F g^−1^ at current densities of 5 and 10 A g^−1^, respectively. These values are lower than the G/Ni nanocomposite, as shown in Fig. 5(b).

**Figure 6:**
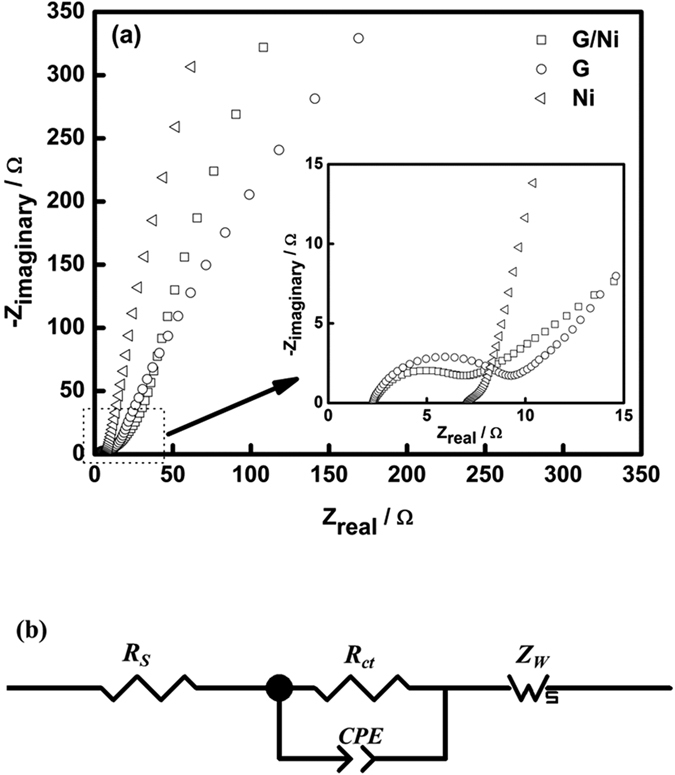
(**a**) Nyquist plots for graphene, nickel nanoparticle and G/Ni nanocomposite and (**b**) proposed equivalent circuit for impedance plot of G/Ni nanocomposite.

EIS was performed to gain a better insight into the fundamental behaviour of electrode materials. In Fig. 6(a), the Nyquist plot of a G/Ni nanocomposite showed a straight line in the low-frequency region. This is called the Warburg impedance (*Z*_*W*_) and is related to the diffusive resistance of the electrolyte through the electrode and ion diffusion/transport into the electrode surface. At the high-frequency region, a small semicircle loop was observed; this loop was assigned to the charge-transfer resistance (*R*_*ct*_), indicating a low internal resistance in the electrode^29^. Low internal resistance would reduce the waste of energy in the form of unwanted heat during the charging-discharging process in energy-storing devices^30^. From the plot (inset of Fig. 6(a)), the semicircle of *R*_*ct*_ of G/Ni was smaller than the pristine one. Similarly, the ESR of a G/Ni nanocomposite was found to be ~2.6 Ω which was lower than that of bare G (3.6 Ω) and bare Ni (6.0 Ω). Based on these results, an equivalent circuit, shown in Fig. 6(b), was proposed to fit the impedance spectra of the G/Ni nanocomposite. Hereby, *R*_*S*_ was the same quantity with ESR, and the constant phase element (*CPE*) was about 0.91.

We have shown that the G/Ni nanocomposite enhanced the capacitance and power density of the supercapacitor. The outstanding performances can be explained by the synergistic effect between EDLC and pseudocapacitance materials. G is an attractive option as an electrode for supercapacitors due to its high specific surface area. The individual sheet of G does not rely upon the distribution of pores in a solid to provide for its extensive surface area. Therefore, it can be used in a simple approach to the surfaces of G materials by the electrolyte while maintaining the overall high electrical conductivity for such a network^31^. The high surface area of G and Ni nanoparticles provides better access of electrolytes by facilitating OH^−^ soaking in bulk materials. In addition, the following reduction/oxidation reaction occurs during the charge and discharge cycle^32^.


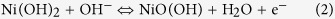


The transportation of electrical charges occurs via a redox reaction of Ni ions between the 2^+^ and 3^+^ chemical states within the nanoparticles and also absorption/desorption of charges on the G surface^33^. Hereby, the dual charge-storage mechanism benefits the capacitance of the G/Ni nanocomposite. Moreover, the G in the G/Ni nanocomposite acted as a conductive support and promoted fast ion and electron transportation, resulting in a rapid charge and discharge process. Since the electrical conductivity of most metal is poor, using G–metal nanocomposites represents a promising way to increase the conductivity and further improve the power density.

The method used to synthesize G/Ni nanocomposites provided excellent specific capacitance and cycle performance at an extensively higher charge/discharge current. Among the available techniques for preparing nanocomposites, ball-milling had several significant advantages, including: simple, direct reaction routes; low-temperature technologies; safer handling reagents and a bulk preparation technique^34^,^35^. Furthermore, ball-milling increases the powder reactivity as well as the surface area of the nanocomposite due to the formation of an interface between reactants^36^,^37^. Consequently, the morphology and structure of a G/Ni nanocomposite is controllable and the nanocomposite is synthesized without impurities. Ni nanoparticles act as spacers that keep the bundle of G nanosheets from restacking. G tends to form agglomerates and re-stack in multilayers through van der Waals interactions^38^ and this problem can be prevent by the introduction of spacers in the interlayer^39^. In this case, Ni as a pseudocapacitance material display high electrical conductivity and electrochemical stability^40^,^41^, can be used as a spacers by increasing the interplanar spacing and thus, making both sides of the G accessible to the electrolyte^42^. G provides a high surface area for the nucleation of Ni^43^. Therefore, this G/Ni nanocomposite can be potentially developed as an electrode for a supercapacitor.

## Conclusion

In conclusion, we have demonstrated a new hybrid nanocomposite for use as an electrode for supercapacitors via a simple approach. G/Ni nanocomposites were prepared successfully using a mechanical ball-milling method. X-ray diffraction (XRD), scanning electron microscopy (SEM), transmission electron microscopy (TEM) and thermogravimetric analysis (TGA) were performed to identify the phases, structures, and morphology of the nanocomposite products, as well as the percentage composition of the nanocomposites. XRD patterns revealed diffraction peaks corresponding to GO, G, Ni and G/Ni nanocomposites. The nanocomposites were successfully synthesized without any impurities. According to SEM and TEM observations, the Ni nanoparticles were circulated in the form of individual particles having a size of approximately 26 nm. Based on TGA, the amount of Ni in the G/Ni nanocomposite was estimated to be approximately 34 wt%. The electrochemical characterization of these electrodes was carried out using cyclic voltammetry (CV), galvanostatic charge/discharge and electrochemical impedance spectroscopy (EIS). The CV curves of the G/Ni nanocomposite had near-rectangular shapes even at a high scan rate of 100 mV s^−1^ indicating good capacitive behaviour. Hybridizing G and Ni nanoparticles as a nanocomposite resulted in the formation of a network on the electrode surface; this resulted in better capacitance that reached 275 F g^−1^ as calculated from the galvanostatic charge/discharge curve at a current density of 2 A g^−1^ in 1 M KOH electrolyte. This is due to a synergistic effect between G and Ni nanoparticles, as well as the advantages contributed from G including high specific surface area and high electrical conductivity. EIS analysis showed that the good conductivity of G/Ni nanocomposites contributed to the low ESR compared to G and Ni. This simple approach can be applied to the synthesis of other G-metals electrodes to upgrade current supercapacitors.

## Additional Information

**How to cite this article**: Mohd Zaid, N. A. and Idris, N. H. Enhanced Capacitance of Hybrid Layered Graphene/Nickel Nanocomposite for Supercapacitors. *Sci. Rep*. **6**, 32082; doi: 10.1038/srep32082 (2016).
